# The Immune-Chemokine Axis in Alzheimer’s Disease: Roles of Adaptive Immune System in Neuroinflammation and Disease Progression

**DOI:** 10.3390/biom16060855

**Published:** 2026-06-11

**Authors:** José Joaquín Merino, José Julio Rodríguez-Arellano, Xavier Busquets, Isabel Álvarez-Vicente, María Eugenia Cabaña-Muñoz, Ana Isabel Flores, Adolfo Toledano Gasca

**Affiliations:** 1 Facultad de Farmacia, Departamento de Farmacología, Farmacognosia y Botánica, Universidad Complutense de Madrid (UCM), 28040 Madrid, Spain; josejmer@ucm.es; 2 Instituto Pluridisciplinar, Universidad Complutense de Madrid (UCM), 28040 Madrid, Spain; 3 Grupo de Medicina Regenerativa, Instituto de Investigación Sanitaria Hospital 12 de Octubre (imas12), 28041 Madrid, Spain; aflores@h12o.es; 4 Functional Neuroanatomy Group, IKERBASQUE, Basque Foundation for Science, 48009 Bilbao, Spain; j.rodriguez-arellano@ikerbasque.org; 5 Department of Neurosciences, Medical Faculty, University of the Basque Country (UPV/EHU), 48940 Leioa, Spain; 6 Laboratory of Molecular Cell Biomedicine, Department of Biology, Faculty of Medicine, University of the Balearic Islands, 07122 Palma de Mallorca, Spain; xavier.busquets@uib.es; 7 Instituto Cajal, Consejo Superior de Investigaciones Científicas (CSIC), 28002 Madrid, Spain; miavmasueco@hotmail.com; 8 Centro de Rehabilitación Oral Multidisciplinaría (CIROM), 30001 Murcia, Spain; mecjj@clinicacirom.com

**Keywords:** amyloid-β, glial activation, T cells, monocytes, neuroinflammation, immunology, neuroimmunology, photobiomodulation, Alzheimer’s disease and neurodegenerative diseases, immunobiology, CXCR6, CXCL0, neuromodulation, neuroinflammation, inflammation, neurology, innate immune system, chemokines, regulation of microglia activation, CX3CR1/fractalkine, T cells, Treg, adaptive immune system

## Abstract

Alzheimer’s disease (AD) is a multifactorial neurodegenerative disorder characterized by amyloid-β (Aβ) and the accumulation of tau in the brain, which triggers robust innate immune responses. Growing evidence indicates that neuroinflammation contributes to AD progression by overactivating microglia through the release of cytokines and chemokines. In general, chemokines can disrupt neuronal communication and promote blood–brain barrier permeability. Peripheral immune cells are mobilized into the brain by a gradient of chemokines. These processes link peripheral immune responses with substantial T-cell infiltration into the CNS parenchyma, leptomeninges and cerebrospinal fluid of both AD mice and AD patients. This finding underscores the relevance of the adaptive immune system, particularly T and B cells, in AD neuropathology. T-cell infiltration into the brain can influence amyloid clearance through chemokine signalling. However, chemokines play a critical role in AD by either promoting or suppressing disease progression. The infiltration of peripheral T and B cells into the brain parenchyma can exacerbate neuronal loss, yet it may also exert neuroprotective effects. Despite the presence of CD4^+^ and CD8^+^ T cells in postmortem brains of AD patients, debate continues about their role in AD brains, in terms of whether they are protective or detrimental. Understanding the complex role of chemokines in controlling innate and adaptive immune responses by modulating neuron–glia interactions (involving astrocytes and microglia) may provide novel therapeutic approaches for AD. Targeting chemokine signalling or treating with drugs that can prevent the recruitment of immune cells may be promising strategies for treating AD neuropathology. Therapies that prevent the overactivation of T cells in the brain could lead to protective strategies against AD. In fact, regulatory T cells (Tregs) could delay the onset of cognitive symptoms, because they suppress inflammation and slow the accumulation of Aβ plaques and p-Tau in the brain. Complementary strategies, such as photobiomodulation, nanoparticle, and T-cell-based approaches, could mitigate AD progression in patients.

## 1. Introduction

AD is a progressive neurodegenerative disorder characterized by the accumulation of β-amyloid (Aβ) plaques and hyperphosphorylated p-Tau in the brain, inflammation, microglia overactivation (the brain’s resident immune cells), astrogliosis, and cognitive decline. Chemokines are inflammatory mediators that induce the recruitment of immune cells into the brain parenchyma. These chemokines act as modulators of the adaptive and immune responses by resident astrocytes and microglia cells. These chemokines are expressed by several cell types in the brain, including neurons, astrocytes, microglia, and vascular cells. In general, the high mobilization of peripheral immune cells into the brain, metabolic alterations and mitochondria dysfunction, and the breakdown of blood–brain barrier (BBB) integrity contributes to AD neuropathology [1,2,3]. Meanwhile, ApoE4 triggers inflammation in the brain, ultimately leading to the overactivation of microglia and astrocytes, as well as T cell activation in the brains of AD patients [4].

In general, chemokines have neuroprotective effects, yet they also contribute to neurodegeneration in rodent models of AD [5,6,7,8,9,10,11,12,13,14,15]. Under controlled inflammatory conditions, microglia can promote neuroprotection. However, chemokine-triggered recruitment of peripheral immune cells (e.g., neutrophils, Treg regulatory cells, T and B cells, and Natural Killer (NK)) exacerbates Aβ and tau deposition, and ultimately induces cognitive dysfunction in AD models [6,16,17,18,19,20]. The infiltration of CD4^+^ and CD8^+^ T cells into the brain can overactivate microglia, though it can also induce neuroprotective effects [18,19,20,21].

From a therapeutic viewpoint, treatments with chemokine antagonists, immune-modulatory drugs that promote Treg-induced responses, or complementary approaches such as photobiomodulation (PBM) are promising strategies for treating AD in patients [22].

This review summarizes the roles of the innate immune system, mainly consisting of microglia and infiltrating monocytes, and the adaptive immune system, consisting of T and B cells, in AD pathology, with a particular focus on chemokines.

We also point out significant gaps in the field regarding drugs that can enhance the removal of amyloid-β plaques in AD patients. Another unsolved issue is the lack of standardized protocols involving chemokine antagonists in AD clinical trials. Furthermore, some clinical studies with AD patients have not evaluated the impact of confounding factors, such as age, infections, and comorbidities. In addition, the long-term adverse effects of antigen-specific CD4^+^ T-cell-based nanodelivery therapies in AD patients remain to be elucidated. Finally, the use of PBM treatment as a complementary therapy requires more clinical evidence in AD patients.

## 2. Role of Innate Immunity in Alzheimer’s Disease (AD)

The Aβ40 and Aβ42 isoforms trigger chemoattractant responses via chemokines [4]. As the brain’s resident immune cells, microglia release chemoattractants (e.g., CCL2, CXCL8, and CXCL10) that recruit peripheral immune cells to the plaque sites in the brain. These chemokines promote the phagocytosis of Aβ by microglia. However, when these microglia cells become exhausted or overstimulated in the brain of AD models, they trigger a cytokine storm that leads to chronic inflammation [23,24]. In transgenic AD models, there is a massive accumulation of highly ramified microglia cells surrounding amyloid plaques. Aβ plaques release chemokines, such as CCL2 (MCP-1) and CXCL8 (IL-8), thereby propagating local inflammation [25]. Thus, chronic inflammation over time impairs the phagocytic capacity of microglia surrounding Aβ plaques over time [13]. Contradictory findings suggest that soluble fractalkine, a chemokine released by neurons, plays a role in preventing the overactivation of microglia in a CX3CR1-dependent manner, since accurate soluble fractalkine maintains correct neuron–microglia interactions. However, low levels of neuronal fractalkine or altered CX3CR1 signalling in microglia cells impair neuron–microglia communication and lead to neurodegeneration in AD models [25]. These chemokines act as “homing” signals for microglia and function as either suppressors or inducers of pathogenic alterations through multiple signalling pathways in the AD brain. Thus, certain chemokine ligands (e.g., fractalkine) can regulate chemotaxis of microglia via CX3CR1 receptors, which sense gradients and drive migration toward amyloid-beta plaques in the brain [7].

A recent study examined the generation of APP/PS1 transgenic AD mice that express thymidine kinase under the control of the CD11b promoter for the selective targeting of microglia. Surprisingly, the ablation with loss of 95% of microglia in these APP/PS1 mice did not alter amyloid pathology. This suggests that microglia are not the primary drivers of Aβ clearance and that other factors may be sufficient to remove plaques [26].

**Figure 1:**
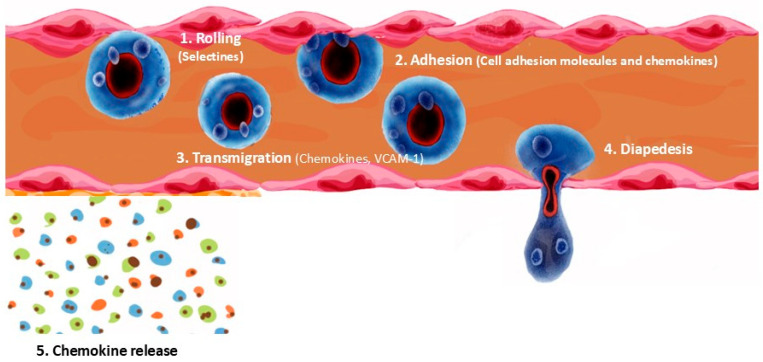
Rolling and migration is directed by chemokines. Interaction between chemokines and cell adhesion molecules involved in BBB disruption in the brains of AD models and AD patients (figure of own elaboration).

Conversely, the recruitment of inflammatory mediators to the brain is directed by chemokines, cell adhesion molecules (e.g., selectins, VCAM-1, and ICAM-1), and integrins (e.g., LFA-1, VLA-4, and α4β1) [27]. Chemokines regulate BBB integrity, which is often compromised in AD patients. Augmented CCL2 and CCL5 levels can weaken BBB tight junctions, allowing peripheral immune cells such as T cells and monocytes to infiltrate into the brain parenchyma. This exacerbates local neuroinflammation and increases neuronal loss. The binding of chemokine ligands to endothelial cells triggers signalling pathways and leads to the reorganization of tight junction proteins. Thus, BBB integrity depends on tight junctions (TJs) that seal the spaces between endothelial cells through interactions among zonula occludens-1 (ZO-1), claudins, and occludins. Consequently, the “zipper-like” seal between cells loosens and allows small molecules to reach the brain parenchyma. Additionally, chemokines induce matrix metalloproteinases (MMPs 2 and 9) and facilitate BBB breakdown by activating astrocytes, microglia, and endothelial cells. Thus, MPPs degrade the basement membrane and the extracellular matrix, while chemokines stimulate the expression of adhesion molecules (i.e., ICAM-1 and VCAM-1) on the endothelial surface. These molecules act as anchors for leukocytes (see Figure 1) [28].

### 2.1. The Role of Innate Immune Cell Types (Monocyte, Neutrophils, and Dendritic Cells) in AD

The role of the adaptive immune system in AD models is not well understood. However, several pieces of evidence suggest that perivascular macrophages (PVMs) may contribute to the clearance of Aβ deposits in the brain. Supporting this hypothesis, PVMs can remove Aβ plaques from cerebral blood vessels in models of cerebral Aβ angiopathy [29]. However, another study argues against a significant contribution of peripheral immune cells to the phagocytosis of amyloid plaques [8]. Microglia and infiltrated macrophages share many surface markers, but PVMs maintain vascular integrity and reduce cerebral amyloid angiopathy. Thus, targeting PVM activation may be a potential therapeutic strategy for clearing vascular amyloid [30]. In another study with bone marrow chimeric mice, the blood-derived monocytes were preserved and microglia were ablated. However, replacing the microglia with peripherally derived cells did not alter the clearance of amyloid [31]. Additionally, the recruitment of bone marrow derived cells is almost absent in a parabiosis mouse model [32]. Therefore, potential therapeutic strategies that aim to regulate the recruitment of monocytes/perivascular macrophages could increase the removal of senile plaques in AD models.

#### 2.1.1. Monocyte Infiltration and Chemokines in AD

The protective or detrimental role of monocyte infiltration in AD is still debated [33]. Postmortem studies confirm the accumulation of CD14^+^ CD16^+^ monocyte-derived macrophages near sites affected by cerebral Aβ angiopathy. During the early stages of AD, macrophages exert neuroprotective effects through TREM2/CD36-dependent phagocytosis of soluble Aβ species. However, Aβ is not effectively degraded [34]. For instance, the CXCR2/CXCL8 chemokine axis, which is released by macrophage/monocytes and other cell types, can activate microglia in an injured brain [35]. In this context, macrophages adopt a disease-associated metabolic profile (DAMP), which is characterized by impaired mitophagy in AD patients [36]. The negative association reported between the low phagocytic activity of peripheral monocytes and the accumulation of Aβ and p-Tau proteins suggests that monocytes and microglia play a role in clearing amyloid beta in AD models [37,38]. In fact, circulating monocyte counts may serve as a biomarker for diagnosing AD. For instance, CCL2 (also known as MCP-1, or monocyte chemoattractant protein-1), CXCL8 (IL-8), CXCL10 (IP-10), and CCL5 (also known as RANTES, or regulated on activation, normal T-cell expressed and secreted) are chemokines that enhance the recruitment of peripheral immune cells [39,40]. In a recent study, researchers compared tau levels between patients with AD (*n* = 127) and 100 age- and sex-matched controls (without neurodegeneration). Their findings confirmed that decreased blood monocyte counts correlated with CSF and plasma tau levels [34,41]. Thus, peripheral monocytes can enhance Aβ clearance, and infiltrating monocytes in the brain may have a greater capacity than resident microglia to remove amyloid plaques [30]. However, in other studies, macrophages have been shown to increase microglial activation and exacerbate neuroinflammation [42]. Finally, a recent study of human cortical organoids confirmed that interleukin-1β (IL-1β) regulates the interaction between neurons and astrocytes. In addition, the CCL3 chemokine was found to increase monocyte infiltration into the parenchyma [43].

#### 2.1.2. Neutrophil-Induced Migration in AD

Aβ deposits in the brains of transgenic AD models induce neutrophil activation. The expression of pro-inflammatory genes, such as NLRP3 and IL1β, appears to be necessary for neutrophil mobilization and cognitive decline in 3xTg AD mice. Furthermore, neutrophils from young AD mice exhibit impaired Aβ phagocytosis and elevated cytokine production in vitro [44]. Conversely, neuroprotective effects were demonstrated in AD models when neutrophil migration into the brain was blocked. In this context, lipocalin-2 (also known as neutrophil gelatinase-associated lipocalin, or NGAL) is a 25 kDa secreted glycoprotein that regulates the innate immune system and induces neuroinflammation in AD by recruiting neutrophils [45]. LCN2 receptors are expressed in neurons, astrocytes, and microglia, and contribute to migration, differentiation, and cell death. Thus, lipocalin-2 represents a potential therapeutic target, and its levels increase in the plasma and postmortem brains of AD patients [45,46,47,48].

#### 2.1.3. Dendritic Cells and AD

Oligomeric forms of human amyloid-β [49] inhibit antigen presentation [50] and promote the differentiation of pro-inflammatory human myeloid dendritic cells. They act as immunomodulators in human-derived dendritic cells (DCs) [51]. CCL3 (also known as MIP-1α) is a potent chemokine that activates both innate and adaptive immune responses. It promotes the recruitment of dendritic cells to lymph nodes and induces T-cell responses [52]. Several cell types, including lymphocytes, macrophages, and dendritic cells, produce CCL3. In addition, CCL19 and CCL21 ligands bind to the receptor CCR7, which controls dendritic cell mobilization and regulates the homing of naïve T cells and mature DCs to lymphoid tissues. Furthermore, monocytes and DCs release CCL18, which attracts T cells under inflammatory conditions [52].

## 3. Chemokine Signaling in AD

Studies in several transgenic mouse models of AD have demonstrated the critical role of chemokines and their receptors in neuroinflammatory processes and AD neuropathology [7]. During an inflammatory state, non-resident peripheral immune cells are recruited into the AD brain through chemokine gradients, thereby amplifying neuroinflammation [6,11]. Certain chemokines, such as CXCL8, and their receptors, such as CXCR2, are elevated in the serum and cerebrospinal fluid (CSF) of patients with AD. These chemokines are present in dystrophic neurites in AD brains [4,7]. Similarly, CCL2 deficiency and CCR2 deficiency leads to Aβ accumulation due to impaired clearance of amyloid [53]. APP-CCR2^−^/^−^ mice, for example, exhibit increased Aβ accumulation, indicating that CCR2 deficiency accelerates the early progression of AD pathology [54]. Consistently, CCR2 deficiency in APP/PS1 mice has been associated with impaired memory performance and enhanced Aβ deposition [55]. Additionally, CXCR2 deficiency in APP/PS1 mice has been shown to reduce Aβ accumulation while increasing the levels of γ-secretase substrates [56].

Taken together, these findings support the hypothesis that altered chemokine expression is associated with impaired Aβ clearance and the progression of inflammation and neurodegeneration. The CCR3/CCL24 axis plays a significant role in AD pathology, and high expression of CCR3 and its ligands is associated with Aβ deposition, gliosis, synaptic impairment, and cognitive decline [57]. Additionally, reduced CCL1 expression has been reported in APP/PS1 mice [58], and this downregulation may contribute to increased Aβ accumulation [59]. Additionally, decreased CCR5 expression has been linked to astrocyte activation and elevated Aβ deposition [60]. On the other hand, CX3CR1 deficiency in AD mouse models, including APP/PS1, and CRND8 mice, has been shown to reduce Aβ deposition by enhancing the phagocytic activity of microglia [61,62]. In addition, CCR5-positive microglia have been identified in association with Aβ deposits in patients with AD [63]. Consistent with this observation, CCR5^−^/^−^ mice exhibit Aβ accumulation and BACE1 expression compared with control animals, suggesting a potential role for CCR5 in removing amyloid plates [64]. Additionally, CXCR3 deficiency reduces plaque accumulation in APP/PS1 transgenic mice and promotes overactivation of microglia [65]. In addition, CXCL10 has been reported to co-localize with Aβ plaques in APP-transgenic mice and has been associated with increased neuroinflammatory responses in AD [66]. In fact, several chemokines, including CCL19, CCL20, CCL24, and CCL27, have been associated with amyloid pathology and neuroinflammatory processes in APP/PS1 transgenic mice [57]. Furthermore, CCR7 deficiency has been linked to impaired glymphatic clearance, enhanced neuroinflammatory responses, increased Aβ accumulation, and cognitive decline in AD mouse models [67]. Studies in AD models using germ-free mice have demonstrated that induced neuroinflammation by microglia and astrocytes depends on the gut microbiome composition. The CXCR4/SDF-1α axis regulates gut dysbiosis in AD because gut microbiota-derived metabolites are released into the bloodstream and can cross the blood–brain barrier, leading to the overactivation of microglia [68]. In AD rodent models, this evidence is supported by the enhanced migration of immune cells from the gut to the brain via SDF-1 α-CXCR4-chemotaxis [69]. Table 1 summarizes the functions of selected chemokines by role.

## 4. Contribution of T- and B-Cell-Mediated Adaptive Immune Responses in AD

In a healthy brain, T-cells, the primary “soldiers” of adaptive immunity, are largely kept out by the BBB, while the leptomeninges represent a site of substantial clonal expansion of CD8 T cells [16]. In AD, inflammation drives the infiltration of cytotoxic T cells into the brain, as BBB disruption increases the number of CD8^+^ (cytotoxic) and CD4^+^ (helper) T cells that enter the brain. These findings are consistent with the elevated number of T and B cells found in the cerebrospinal fluid of AD patients [70]. However, several lines of evidence suggest that cerebral Aβ deposition alone is insufficient to drive T-cell infiltration. Another study indicates that antigen presentation at perivascular sites facilitates the infiltration of CD8^+^ T cells into the brain parenchyma. Additionally, other proteins such as leucocyte function-associated antigen (LFA-1) contribute to T-cell infiltration in AD brain [27], as do ICAM-1 and VCAM-1 on endothelial cells in AD brains [28]. This suggests that Aβ-induced endothelial activation may contribute to T-cell infiltration in AD [34].

Three hypotheses may explain the link between T-cell dysfunction in AD.

### 4.1. Hypothesis of Altered T-Cell Function in the AD Brain

Three hypotheses link T-cell dysfunction to AD—viral reactivation, increased chemokine-driven brain T-cell recruitment, and infection-induced T-cell exhaustion—all of which promote neuroinflammation [70,71,72,73]. T cells are activated by antigen-presenting cells and support immune surveillance. However, autoreactive T cells in the brain can amplify neuroinflammation [74,75]. T cells, macrophages, and microglia increase the clearance of extracellular amyloid protein [20,30,37], while T-cell overactivation induces neuronal death [75]. In aging, T cells undergo immunosenescence. CD8^+^ TEMRA cells become exhausted, cytotoxic, and sometimes NK-like and contribute to AD pathology [20,75].

On the other hand, the dura mater drains soluble antigens released into the CSF through the brain’s glymphatic system [16]. In mice, meningeal macrophages internalize CSF-derived proteins and present antigens to T cells as antigen-presenting cells (APCs) [70,76]. Lymphatic function is reduced and neuroinflammation is exacerbated in the meninges of aged mice [77]. In mouse models, the meninges regulate inflammation [70] and microglia recruit and present antigens to T cells in tauopathies [78]. In the CNS, T cells and mature APCs contribute to the immune surveillance (https://www.sciencedirect.com/topics/immunology-and-microbiology/immunosurveillance, accessed on 26 February 2026) in perivascular and meningeal spaces [79,80]. T cells retained in the brain parenchyma are often autoreactive T cells and amplify neuroinflammation [73,81]. Conversely, in the absence (https://www.sciencedirect.com/topics/neuroscience/epileptic-absence, accessed on 26 February 2026) of antigen-specific activation, T cells are “naïve cells”. T cells interact with APCs that express the major histocompatibility complex (https://www.sciencedirect.com/topics/neuroscience/major-histocompatibility-complex, accessed on 26 February 2026) (MHC) after entering circulation. Through their T-cell receptors (TCRs) (https://www.sciencedirect.com/topics/biochemistry-genetics-and-molecular-biology/t-cell-receptor, accessed on 26 February 2026), these APCs can present T cells with virus antigens or self-antigens, causing naïve T cells to transform into effector and memory T cells (https://www.sciencedirect.com/topics/biochemistry-genetics-and-molecular-biology/memory-t-cell, accessed on 26 February 2026) that release pro-inflammatory cytokines [82].

In addition, the local immune environment within the leptomeninges modulates T-cell clonal expansion, as well as direct Natural Killer (NK) cell-mediated killing of activated CD8 T cells [83]. These NK cells may regulate the number of CD8^+^ T cells at the vessel lumen in the human leptomeninges by actively limiting their numbers through direct contact and targeted killing [83]. Interestingly, AD patients with high leptomeningeal CD8 T-cell resident memory (TRM) clonal expansion have less inflammation. This suggests that a deficiency of clonally expanded CD8 T_RM_ cells in the leptomeninges enhances neuroinflammation in AD [70]. Meningeal NK cells also influence astrocyte-mediated killing of infiltrating lymphocytes through cytokine production [84]. These CD8 T cells are the primary source of clonally expanded T cells in the leptomeninges and are potentially regulated by microglia and local leptomeningeal; CD8 regulatory T cells to control neuroinflammation in AD [85,86]. Thus, peripheral innate immune cells are critical modulators of AD pathogenesis [75]. In addition, infiltrated macrophages in the brain of AD models have a greater ability than resident microglia to remove CNS plaques [87]. Infiltrating T cells can interact with damaged resident astrocytes and neurons, leading to overactivation of microglia in AD models [88]. Consistent with these findings, a recent report confirmed increased T-cell clonal expansion in the CSF of patients with mild cognitive impairment associated with aging and late-onset AD [77,89]. In conclusion, the infiltration of peripheral T and B cells into the brain parenchyma can exacerbate neuronal loss [89], yet it can also have neuroprotective effects [90]. Shortened telomeres in T cells can explain the detrimental role of adaptive T and B cells in AD neuropathology [91]. Additionally, TNF-α-induced apoptosis by T cells in AD patients and lower CD28 expression of CD8^+^ T cells correlated with AD severity [92]. Another study confirmed a decreased percentage of naïve CD4^+^ T cells and a high percentage of terminally differentiated memory CD4^+^ T cells, with no change in CD8^+^ T-cell phenotypes. In these AD patients, more senescent phenotypes were restricted to CD4^+^ T cells [93]. Additionally, leptomeningeal CD8 T-cell clonal expansion and T-cell activity are impaired by Aβ-reactive T cells in AD [94]. The reduced protective effects of T-cells are explained by the low number of mature APCs in the brains of APP-transgenic mice; most of the APCs in amyloid-burdened brains are immature. T cells are activated by mature APCs, as well as by activated macrophages and antibody-producing B cells in CD4^+^ T-helper cells [72]. However, amyloidosis could impair the presentation of antigens by directly blocking T-cell activation in AD rodent models [80]. A recent study has revealed that the ApoE ε4 genotype alterations are associated with peripheral T-cell dysregulation, as overt T-cell activation induces neuronal death [94,95].

Additionally, the release of IL-17 and IFN gamma by overactivated microglia (Th1 and Th17 responses) leads to detrimental effects in T cells and neurodegeneration in AD [95,96]. Furthermore, CD8^+^ T cells can attack neurons and exacerbate amyloid-related cognitive impairment [96], while IL-17-dependent responses associated with γδ T cells promote cognitive impairment at the early stages of AD neuropathology [97]. The high frequency of Th17 cells indicates that the activation of adaptive immune cells is associated with neurodegeneration in AD [98]. T cells are the primary source of interferon γ (IFN-γ)-producing cells, and neurons and microglia express IFN-γ receptors [99]. Thus, a lack of T-cell-derived IFN-γ responses could impair phagocytosis by microglia and macrophages [100]; in fact, IFN-γ enhances the phagocytosis of Aβ-immunized APP-transgenic mice [94]. In this context, certain chemokines (CCL2, CCL5, CXCL10, CXCL16) in IFN-γ-stimulated microglia and astrocytes can enhance the mobilization of immune cells from the periphery to the brain [97]. IFN modulates microglial activation and antigen presentation in the brain [98,99]. Microglia activation downstream of CD8+ T-cell signalling can promote acute or chronic inflammation in the AD brain [99,100,101,102]. Therefore, it is reasonable to assume that certain levels of IFN-γ are necessary for an adequate immune response in AD. Conversely, the anergic role of T-cells might inhibit the phagocytic activity of microglia by releasing anti-inflammatory mediators, such as IL-10, in AD models [103]. However, cytotoxic T-cell-mediated responses are absent in the APP-transgenic AD mice because the brain does not orchestrate T-cell-induced protective responses against amyloid-beta peptide toxicity [104]. Consistent with this evidence, T-cell-induced cytotoxic responses are absent in human AD brains [104]. Therefore, identifying risk factors that contribute to AD pathology in the blood and CSF of AD patients could help to develop better pharmacological interventions. Collectively, these protective T-cell-induced effects are mediated by the phagocytosis of extracellular amyloid plaques by macrophages or microglia [105].

### 4.2. The Role of CD8^+^ TEMRA Cells in AD

Experimental evidence from rodent models of AD suggests that depleting mature B cells reduces neuroinflammation and cognitive decline by decreasing the presence of DAM molecules [106]. DAMs promote chronic inflammatory signals and lead to dysfunctional microglia, which impairs neuronal function [106,107,108]. Growing evidence indicates a role of CD8^+^ tissue-resident memory T cells (Trm) in AD progression. In particular, high levels of CD4^+^ and CD8^+^ TEMRA cells are specifically detected early in the disease. CD4^+^ TEMRA cells, but not CD8^+^ TEMRA cells, positively correlate with AD biomarkers, such as the serum Aβ_42/40_ ratio, Nfl, and GFAP [108,109,110,111,112]. This suggests that TEMRA cells increase T-cell functionality at the early stages of AD neurodegeneration [113]. Therefore, strategies that can block or reshape T-cell responses are promising for mitigating neurodegeneration [114].

CD8^+^ T-cell subsets are a new area of research for AD. These cells drive microglial function [112,113,114] and the recruitment of brain T cells, which provokes changes in T-cell receptors (TCRs) and increases neuroinflammation [115]. Conversely, CD8^+^ T cells are induced by peripheral B cells in the human AD brain and act by targeting disease-associated microglia (DAM). Ablation of these CD8^+^ T cells and B cells block amyloidosis-linked neurodegeneration and decreases CD8^+^ T-cell accumulation in the brain, making them therapeutic targets for controlling AD [112,116]. A recent study found that enhanced amyloid clearance and lower Aβ burden were associated with increased phagocytic activity of microglia in APP-PS1-dE9 transgenic mice lacking mature B and T cells due to peripheral T-cell depletion in this model [117]. Changes in T-cell reactivity to the Aβ peptide in the early stages of AD have been reported in patients with AD. In fact, analysis of the single-cell transcriptional profiles of CD4^+^ and CD8^+^ T-cell populations in peripheral blood suggests impaired CD4^+^ regulatory T-cell function in subjects with early AD pathology [118]. Patients with minor cognitive impairment showed signs of neurodegeneration associated with less immunosuppressive Treg responses and enhanced pro-inflammatory CD8^+^ TEMRA/effector transcriptomic profiles in AD patients [74,118]. In these patients, autoreactive CD4^+^ memory T cells were reported against the Aβ_1-42_ peptide, independently of latent widespread *Herpes viridae* infection [118,119]. T cells could contribute to AD pathology in APP23-transgenic (APP23-tg) mice, as T-cell populations, particularly CD8^+^ T cells, are located around Aβ plaques, during the late stages of AD with amyloid. These CD8^+^ T cells indirectly exacerbate neurodegeneration by regulating IFN-γ-associated signaling in glial cells [99,102,120].

The presence of T-cell populations, particularly CD8^+^ T cells, has been demonstrated in late disease stages of APP23-transgenic AD mice, clustering around Aβ plaques. Accumulation of CD3^+^ and CD8^+^ T cells has been reported in the brains of APP^NL-G-F^ transgenic AD mice and contributes to neurodegeneration [121]. Furthermore, activation of microglia downstream of CD8^+^ T-cell signalling can lead to a chronic inflammation in the brain [102,108]. However, T-cell-derived IFN-γ shapes the response of microglia rather than T-cell recruitment per se [102]. Furthermore, the Aβ plaque-associated subset of CD8^+^ T cells that express interferon-stimulated genes (ISGs) contribute to amyloid-related neuroinflammation [120]. Thus, type-I interferon IFN microglia cells near plaques contribute to the progression of amyloid pathology in AD patients and T-cell-mediated neuroinflammation. Specifically, this population of microglia enhances synapse loss in human AD brains [120]. Additionally, the selective accumulation of CD8^+^ T cells has been reported not only in the brains of both 5xFAD transgenic mice [121] and P301S-prion mice [78], but also these cells contribute to neurodegeneration. Interestingly, brain sections from P301S^+^/^+^ mice with CD8^+^ T cells have less phosphorylated tau than those without CD8^+^ T cells [121]. In another study, TCRα-deficient 5xFAD at 4 and 10 months of age (mice lacking both CD8^+^ and CD4^+^ T cells: 5xFAD; *Tcra^−/−^* mice) exhibited high Aβ deposition in the brain [122]. It is worth mentioning that an increased number of CD8^+^ T cells have been reported in brain areas with tau pathology. In a tauopathy mouse model with neurofibrillary tau tangle pathology, depleting T cells using anti-CD4 and anti-CD8 antibodies mitigated brain atrophy and decreased cognitive dysfunction from 6 to 9.5 months. Moreover, astrogliosis and microglia overactivation decreased by 5 months of age due to the depletion [123].

Conversely, a study found that a decreased number of CD8^+^ T cells leads to increased Aβ plaque deposition. Thus, these findings revealed a protective role of CD8^+^ T cells against AD progression [124], which is supported by decreased Aβ accumulation in the brains of AD mice through the genetic disruption of CD8^+^ T cells. Consequently, chemokine signalling in CD8 T cells could protect against neuroinflammation in AD [125,126].

#### Is There Any Relationship Between Viral Infections and AD?

Recent studies suggest that certain bacterial infections play a role in the brains of AD models. For example, *Borrelia burgdorferi* sensu lato (Bbsl) invades the brain and provokes a persistent neuroinflammatory state that enhances Aβ and tau accumulation by neuroborreliosis (Lyme disease) [127].

Certain viral infections, such as hepatitis B and influenza, are known to trigger chronic inflammation and provoke BBB disruption [127], which leads to Aβ neurotoxicity [128] and memory impairment in AD [129]. In this sense, influenza and herpes viruses can cause neurodegeneration [130]. However, the presence of highly clonally expanded T cells specific to these viruses in the leptomeninges does not confirm that they cause viral AD. The CD8^+^ subtype of T cells has a higher frequency of TEMRA/effector cells, which increases the pro-inflammatory gene expression profile and decreases antigen responsiveness. Nevertheless, the presence of autoreactive CD4^+^ memory T cells against the Aβ_1-42_ peptide is independent of latent, widespread *Herpes viridae* infections in AD patients [74]. Downregulation of genes involved in antigen processing and immunoglobulin binding suggests impaired adaptive immune responses by T cells in AD [123]. Recent studies have shown that T cells expressing TCRs against viral antigens can regulate brain T-cell-mediated autoimmunity via meningeal lymphatics to cervical lymph nodes [77]. Additionally, *Toxoplasma gondii* infection in the brains of 3-month-old 5xFAD transgenic mice was associated with increased monocyte infiltration and a high number of CD4^+^ and CD8^+^ T cells, as well as overactivation of microglia [131]. Another study confirmed that the number of CD8^+^ T cells increased in the brain parenchyma at 10–12 months of age. Improvements in cognitive function associated with reduced Aβ plaque accumulation were observed following CD8^+^ T cell depletion [132,133,134,135,136].

In conclusion, the adaptive immune system in AD, the upregulation of effector T-cell activities [132], and impaired Treg cell-related immunomodulatory mechanisms accelerate the progression of AD in transgenic AD models [133]. Additionally, exposure to certain pollutants (e.g., mercury and aluminum) may accelerate neuroinflammation, and early-life exposure to herpes simplex virus (HSV-1) can provoke cerebral amyloid angiopathy [134].

### 4.3. The Protective Effects of T Cells and Regulatory T Cells (Tregs) Against AD

Treg cells act as the “peacekeepers” by suppressing excessive inflammation and “rejuvenating” and maintaining immune homeostasis. One study suggests that T cells have a neuroprotective role against AD because they may prevent neurodegeneration. Intracranial infusion of the anti-Aβ antibody (3D6) restored inflammation to normal levels by activating microglia in a CCL2-dependent manner [40]. Treg cells mimic T-cell signalling to coordinate the brain’s response to Aβ plaques and maintain complement-activating pathways. Consistent with the protective effects of Tregs, Tregs enhance the phagocytosis (engulfment) of amyloid and tau in 5xFAD and 3xTg-AD models. Additionally, T cell exosomes play a protective role in cells [137,138]. Another study found that transient depletion of Foxp3^+^ Tregs or pharmacological inhibition of their activity was followed by reduced neuroinflammation, enhanced Aβ plaque clearance, and improved cognition. These results suggest that targeting Treg-mediated systemic immunosuppression could be an effective treatment for AD pathology [116].

## 5. Chemokine Signalling and T Cells in AD

### 5.1. The CXCR4/SDF-1α Axis Influences Microglia–T-Cell Crosstalk in AD Models

SDF1 alpha is associated with an increase in brain-associated B-lineage cells that express the CXCR4 receptor. CXCR4^+^ antibody-secreting cells are significantly reduced in the gut of 5xFAD AD mice. Conversely, CXCR4^+^ antibody-secreting cells (ASCs) are present in the colon, while CXCR4^+^ B cells and gut-specific IgA^+^ cells accumulate in the brain and dura mater. These findings suggest that there is enhanced SDF-1α-dependent recruitment of immune cells into the brains of 5xFAD mice. In this model, SDF-1α is released by astrocytes, and its levels correlate with the infiltration of CXCR4^+^ B cells and gut-specific IgA^+^ cells into the brain and dura mater. These effects appear to be SDF-1α specific because CXCR4 blockade by AMD3100 (a CXCR4 antagonist) abolished the migration of immune cells into the brain [69].

### 5.2. The CXCR3 and CXCR6 and Microglia–T-Cell Recruitment in AD Models

Chemokines increase T-cell recruitment in a CXCL10-CXCR3-dependent manner. The CXCR3 chemokine receptor enables the recruitment of specific memory CD8^+^ T cells to areas of the cortex of 5xFAD AD transgenic mice [18] containing activated APCs that express CXCL9/10/11 [139,140].

CD8^+^ T cells contribute to neurodegeneration through direct cytotoxicity and indirect glial-enhanced inflammatory responses. In fact, infiltrated CD8^+^ T in the hippocampus and cortex of 6- to 7-month-old 5xFAD mice express CXCR3 [18,140]. This subset of CD8^+^ T cells associated with Aβ plaques promotes type-I interferon signalling and recruits non-ISG T cells through the CXCR3-CXCL10 axis. Therefore, type-I interferon responses in microglia cells near plaques could be a target for drugs that prevent the accumulation of amyloid plaques in the human AD brain [141]. Additionally, certain chemokines, such as the CCR2/CCL2 axis, recruit leukocytes to the brain and promote the differentiation of naïve T cells [7]. Other chemokines, such as CXCL8, activate neutrophils and T cells in newborn humans and are anti-inflammatory, with implications for immune monitoring and immune interventions [96].

Conversely, Cxcr6 deficiency or CD8^+^ T-cell depletion decreased the clonal expansion of brain PD-1^+^ CD8^+^ T cells in the brain and increased the release of pro-inflammatory cytokines in microglia. In this study, the high number of CD3^+^TCRβ^+^ T cells is due to the accumulation of CD8^+^ T cells [113]. Additionally, CXCR6 plays a role in memory formation. Its deficiency disrupts the spatial localization of CD8^+^ T cells near Aβ plaque-associated microglia, resulting in memory deficits in AD transgenic mice. Once recruited, T cells engage in reciprocal interactions with microglia to reinforce their activation and neurodegeneration [113]. These findings suggest a key role for the CXCR6 chemokine in AD. Indeed, the analysis of single-cell RNA sequencing datasets of brain-derived CD8^+^ T cells confirmed a CXCR6-related immunosuppressive cluster with stem-like features in Cxcr6-deficient AD mice. Since CXCR6-related CD8^+^ T cells decrease Aβ pathology, they may have a neuroprotective role in the brain [80] (see Table 2). Analyzing single-cell RNA sequencing samples from CSF in AD patients, compared with 45 cognitively normal subjects (aged 54–82 years), revealed CXCR6’s role in CD8 T-cell recruitment. In this study, defective CD8 T cytokine signalling was associated with decreased expression of lipid transport genes in monocytes. Taken together, these findings suggest that the CXCL16-CXCR6 signaling axis promotes the recruitment of antigen-specific T-cells into the brain via CD8^+^ T effector memory T cells [142]. Another study of the CSF from cognitively impaired AD patients reported increased CXCR6 expression in CD8^+^ memory T cells, along with increased levels of CXCL16 in microglia and monocytes. These results suggest that this chemokine axis regulates the recruitment of brain-resident T cells into damaged areas of the CNS [76,143].

**Figure 2:**
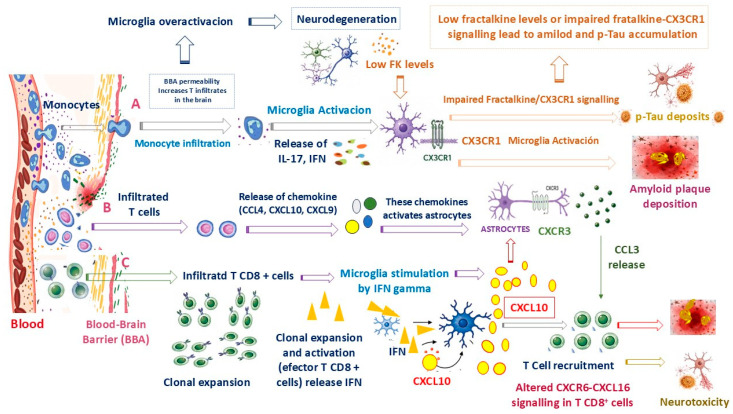
Interactive effects between the innate and adaptive immune responses in the brain of Alzheimer’s patients.

As shown in Figure 2A, the progressive aggregation of amyloid-β in extracellular amyloid plaques and of tau protein in neurofibrillary tangles is a pathological hallmark of AD. Neuroinflammation, neuronal death, synaptic loss, and monocyte infiltration can compromise the blood–brain barrier. These immune cells infiltrate the brain parenchyma and interact with glial cells and neurons. In a rodent model of tauopathy, the neuroinflammatory response by activated microglia and effector-type T cells contributes to neurodegeneration in a CX3CR1-fractalkine-dependent manner. In fact, the release of low levels of soluble fractalkine by vulnerable neurons impairs the function of microglia. This leads to the accumulation of p-Tau and the deposition of amyloid beta in the brains of patients with AD and taupathies [114]. The presence of activated T cells has been demonstrated in the brains of transgenic mice with frontotemporal dementia (FTD), which correlates with overactivated microglia and tau deposition. An increased number of activated T cells (CD4^+^ and especially CD8^+^ cells) are found in the parenchyma, leading to neurodegeneration. Interestingly, tau transgenic mice with an apolipoprotein E (ApoE) knockout background were protected against T-cell infiltration. This suggests that T-cell infiltration is associated with tau pathology progression (Braak staging). Neuronal tau aggregation triggers microglia activation and results in the recruitment, clonal expansion, and activation of T CD8^+^ cells in the brain. This inflammatory cascade is enhanced by microglia MHC-II and CD11c microglia cells, as IFN gamma, released by clonal CD8^+^ cells, activates T cells in the brain. Taken together, these findings link T-cell accumulation with p-Tau and amyloid-beta deposition, resulting in neurotoxicity and neurodegeneration.

As shown in Figure 2B, activated T cells have been found in the postmortem brains of individuals with different neurological disorders, including AD, particularly in regions experiencing neuronal loss and tau pathology, such as the hippocampus and limbic structures. These infiltrating T cells activate astrocytes in the brain parenchyma, prompting the release of chemokines, such as CXCL10, CXCL9, and CCR4 chemokines. Certain chemokines, such as CCR3, which are released by infiltrating T cells, can overactivate microglia, leading to neurotoxicity. This exacerbates local inflammation and enhances neuronal loss.

As shown in Figure 2C, the infiltration of CD4 and CD8^+^ T cells into the brain parenchyma promotes detrimental effects, leading to Aβ plaque accumulation and microglia overactivation through the release of the chemokine interleukin-17 (IL-17) by microglia. In fact, CD8^+^ clonal infiltrates are found in the brain parenchyma, where they activate microglia through the release of IFN gamma from activated CD8^+^ T cells. Overactivated microglia release the chemokine CXCL10, which binds to the CXCR3 chemokine receptor in astrocytes. Thus, CXCR3 contributes to neurodegeneration in conjunction with altered CXCR6-CXCL16 signalling by CD8^+^ T-cell infiltrates in the brain (see Figure 2A–C, as well as Table 2).

Table 2 illustrates the results of studies with T cells in AD, including AD models and AD patients, with an emphasis on the experimental models, immune cell subtypes, key signalling pathways, and protective versus detrimental effects in AD models and patients.

## 6. Gaps and Future Interventions in Chemokine Signaling in AD

There are significant knowledge gaps in the field of chemokines and neuroinflammation in AD. Current knowledge about blood-derived mononuclear cell infiltration in the AD brain parenchyma is limited to animal models. It is unclear whether T-cell recruitment to the brain is a compensatory mechanism against amyloid-β and tau accumulation contributing to cell death, given the dual protective/detrimental role of T cells. Another gap is understanding the role of chemokines in initiating neurodegeneration and the exact molecular mechanism by which chemokines promote a silent chronic, inflammatory state in AD brains by chemokines (e.g., CCL2, CXCL10, and CX3CL1 are augmented in AD patients). Therefore, it is impossible to confirm whether chemokine changes reflect the causality of AD neuropathology. Chemokine systems are pleiotropic ligands, meaning they bind to the different receptors, and vice versa. Thus, blocking a single chemokine receptor could be compensated by different chemokine ligands. This feature could decrease the efficacy of certain chemokine antagonists in clinical trials. Different chemokine signalling pathways depend on the disease state and cell type, as well as neuron–glia interactions in the injured brain. Additionally, chemokines exhibit distinct receptor expression patterns in astrocytes, neurons, and vascular cells, and can regulate microglial activation. For instance, the neuroprotective or neurotoxic role of soluble fractalkine depends on metalloprotease activation and the stage of AD progression [144]. On the other hand, inflammation is a dynamic process that can result in protective responses in early AD, but not in late stages. Additionally, tracking chemokine changes in living patients is limited in longitudinal studies. Furthermore, because chemokine signalling pathways differ between species, clinical trials often fail or are inefficient. Furthermore, the role of chemokine pathways in linking Aβ and p-Tau deposition in the brains of patients with AD is not fully understood. In fact, AD rodent models often overexpress amyloid, yet they do not fully replicate AD progression in patients or translate well to human subjects. Although chemokines are promising biomarkers of AD in CSF and blood samples, their levels vary across clinical trials. Regarding biomarker development, the absence of a standardized panel of chemokines introduces variability in clinical studies. In addition, some studies did not consider confounding factors (age, infections, comorbidities) when interpreting AD diagnoses. Timing, specificity, and patient stratification are major unresolved issues that can affect the inflammatory responses; given the different “inflammatory phenotypes” in AD pathology (e.g., ApoE genotype), the study of single-cell specific chemokine signaling pathways would identify new targets for neuroprotective and anti-inflammatory drugs in AD. Unfortunately, as AD progresses, Tregs often become “exhausted” or dysfunctional, reducing their efficacy. Thus, strategies that increase the number of Treg and their functionality are other protective strategies against AD progression. More research is needed on the use of T cells as a target for drugs in the brains of AD patients before it can be translated into clinical practice.

## 7. Therapeutic Intervention in AD: Effects on Immune and Adaptive Immune Responses

### 7.1. Control of Adaptive Immune Response

Removing microglia or T cells, or suppressing their activation by IFNγ, reverses the neuroinflammatory responses in the brain. This leads to neuroprotective effects against tau pathology [145]. New insights are emerging about how engineered T cells provide protection against AD pathology. A recent study used antigen-specific CD4^+^ T-cell-based nanodelivery cell therapy for AD with no potential side effects. This study found that CD4^+^ Treg cells and microglia contribute to effective immunotherapy against AD, suggesting a protective role of Treg cells. In this cell-based nanodelivery strategy, Aβ1-42-specific, brain-infiltrating CD4^+^ T cells were detected in the brains of APP/PS1 transgenic mice, resulting in neuroprotection, decreased Aβ deposition, and diminished synaptic damage. These protective effects are associated with a greater percentage of Treg cells in the brain, favoring protective plaque-associated microglial responses in AD [145]. Tregs maintain immunological homeostasis and antigen tolerance [146], in both physiological and pathological conditions [147,148]. Interestingly, Tregs play an important role in generating several subtypes of reactive astrocytes in AD, which are linked to amyloid deposits [149]. While Tregs do not directly affect the number or morphology of these astrocytes, early Treg depletion reduces neuroprotective A2 astrocyte-dependent responses [137].

### 7.2. Pharmacological Interventions: Agonists and Chemokine Blockers (Antagonists)

In AD settings, T cells can contribute to disease progression and serve as a potential target for intervention. Immunoassays have also linked CXCL8 to the recruitment of T cells (γδ T, CD8^+^ T), suggesting that it plays a role in sustaining neuroinflammation. Among potential therapeutics, nonsteroidal anti-inflammatory drugs have emerged as modulators of CXCL8-driven pathology [149]. For instance, ibuprofen has been shown to significantly suppress neurodegeneration by regulating CXCL8 expression. Altered CXCL8 signalling impairs neuron–glia communication in the brain, leading to p-Tau deposition in AD models via the p38 MAPK signaling pathway [16]. Since chemokines drive the immune response, they are major targets for CXCR4 antagonists, such as AMD-3100, and CCR2 or CCR5 (maraviroc). Additionally, drugs that activate the CX3CL1/CX3CR1 signaling pathway can prevent neurodegeneration and reduce microglia overactivation [144]. In an AD transgenic model, brain-infiltrating CD8^+^ T cells impair the transition of microglia into AD-associated states and suppress amyloid clearance via CCL5-CCR5 signaling. Interestingly, pharmacological blockade of CCL5 attenuates amyloid deposition, but CCL5 administration exacerbates AD pathology [85].

A recent study suggests that chemokine antagonists or genetic intervention may have a therapeutic value in modulating chemokine signalling. For instance, in a 3D in vitro human neuroimmune model, treatment with MAB160 (an anti-CXCR3-neutralizing antibody) resulted in a dose-dependent pattern of T-cell attraction by CXCL10 [143]. Thus, blocking T-cell trafficking and infiltration with a chemokine blocker may reduce brain infiltration mediated by the CXCL10-CXCR3 and the CXCL16-CXCR6 axes, which stabilize the BBB. At later stages of AD, inhibiting IFN-γ signalling may mitigate CD8^+^ T-cell-mediated neuronal damage. Blocking of the IFN pathway can exert protective or immunoregulatory roles in T cells [143].

### 7.3. Cobrotoxin (CTX) for AD Treatment

Recent studies indicate that CTX is a potent modulator that regulates neuroimmune signalling and mitigates neuroinflammation and synaptic dysfunction in a 5xFAD transgenic AD rodent model. This study observed reduced brain-infiltrated CD8^+^ T cells and downregulation of Cxcl9, Cxcl10, and Cxcl16 chemokine expression after nine weeks of CTX administration. These results suggest that CTX modulates T-cell–microglia communication. In conclusion, CTX, a short-chain neurotoxin derived from *Naja atra* venom, attenuates microglial activation and decreases pro-inflammatory cytokine release while preserving plaque-associated disease-associated microglia (DAM) markers [150].

### 7.4. Treg Cell Activation

In AD, the immunomodulatory mechanisms of regulatory T cells (Tregs) are compromised, shifting the immune system toward an anti-inflammatory response. Recent studies have examined Tregs as potential neuroprotective agents against neuroinflammation in AD. One such study confirmed the safety and efficacy of a low dose of interleukin-2 (IL-2) in expanding Tregs to prevent AD progression without adverse effects (NCT06096090). In this randomized, double-blind, phase 2a study, 38 AD patients received a subcutaneous IL-2 (10^6^ IU/day) for five days. Results were compared with those of a placebo group. IL-2 was administered either every 4 weeks or every 2 weeks, for 21 weeks, followed by 9 weeks of observation. The results confirmed that IL-2 doses increased the percentage of Treg and decreased CCL2, CCL11, and IL-15 chemokine levels while increasing IL-4 and CCL13 levels. Furthermore, there was a significant reduction in CSF Aβ42 levels compared to the placebo group [135]. Treatment with low IL-2 concentration can restore Treg function and reduce Aβ accumulation, both of which are often impaired in AD patients [151]. Finally, Treg-derived extracellular vesicles can exert neuroprotective effects and reduce inflammation (IL-6, IL-2, and IFN-γ) in effector T cells through multiple mechanisms in target cells [88]. Additionally, advanced CAR-Treg therapies are being explored to prevent the formation of protein aggregates in the AD model brains [133].

### 7.5. The C3 Complement Strategy for Preventing AD Progression

Complement proteins play a role in neuroinflammatory responses in AD models. For instance, the C3 factor increases the Abeta Aβ clearance by microglia in the neurons of C3-deficient APP-transgenic AD mice [152]. In fact, infusion of the C3a receptor antagonist induces apathy-like behavior and increases immune-related gene expression in 16-month-old 5xFAD mice [153]. Therefore, targeting complement pathways may be a novel therapeutic strategy for alleviating apathy in AD pathology [154].

### 7.6. Valacyclovir

This anti-inflammatory and neuroprotective drug can increase clearance of Aβ and p-Tau in the brain [155].

### 7.7. Photobiomodulation (PBM)-Based Strategies for Preventing Excessive T-Cell Migration in the AD Brain

PBM transforms photons absorbed by cellular chromophores into photochemical energy, which can mediate biological effects. This leads to changes in intracellular calcium concentrations that contribute to neuronal function [155,156,157]. Several studies using LEDs and lasers in the red to near-infrared (NIR) spectrum have demonstrated the therapeutic potential of PBM against chronic neuroinflammatory processes, mitochondrial dysfunction, and cerebral hypometabolism. PBM can also prevent the overproduction of reactive oxygen species (ROS). In APP/PS1 AD transgenic mice, 6 weeks of non-invasive PBM treatment activates mitochondria energy metabolism and promotes the anti-inflammatory effects of microglia, enhancing Aβ clearance and ameliorating cognitive dysfunction [158]. PBM activates cellular processes by stimulating complex IV of the electron transport chain, which is mediated by cytochrome c oxidase. Under stressful and neuroinflammatory conditions, nitric oxide (NO) levels increase. This can inhibit cellular respiration and reduce ATP production [156,159,160,161]. In fact, PBM using wavelengths in the NIR range, as an adjunct to other treatments, has demonstrated modulatory effects on brain metabolic function, promoting neurogenesis and synaptogenesis. Furthermore, PBM provides neuroprotection by activating anti-inflammatory and antioxidant signalling pathways during brain-directed PBM and LED-based therapies [141,145,146,147,156,161,162,163]. Conversely, the systemic peptide GHK-Cu binds and transports copper to sites of inflammation for tissue repair [164]. GHK-Cu enhances copper bioavailability, enabling cells to synthesize antioxidant enzymes, such as superoxide dismutase (SOD), which helps combat oxidative stress [160,162,164,165]. Additionally, PBM can also modulate transcription factors, such as NF-κB and other signalling mediators. The beneficial effects depend on exposure time, the tissue’s basal redox state, mitochondrial density (particularly high in brain tissue), tissue depth, and wavelength. These factors collectively influence the therapeutic window [113,155,157,160,166]. Furthermore, the photonic influence as a systemic messenger and biological accelerator is a new perspective being investigated as remote photobiomodulation (PBM) that could exert various biological effects. These effects include improving mitochondrial energy metabolism, inhibiting glycolysis, improving mitophagy, mitigating dysbiosis, reducing neuroinflammation by activated glial cells, promoting oxidative phosphorylation, increasing cerebral perfusion, modulating the immune system, and regulating the gut microbiome through myokines [158,162].

Prospects in neurolymphophotonics offer promising opportunities for developing novel therapeutic technologies [142]. Evaluating the role of PBM in the immune system may prevent the progression of AD through various delivery approaches, including transcranial, intracranial, intranasal, oral, and auditory routes [155,166,167]. Therefore, investigating the effects of laser-based therapies on the immune system is a preventive strategy that promotes Aβ clearance via the glymphatic and lymphatic systems through neurolymphophotonics. This process may be enhanced during sleep [155,157,162,163,166,167,168]. Furthermore, sleep disorders are increasingly recognized as potential risk factors for cognitive decline, accelerating neuroinflammatory processes [169]. It has been hypothesized that laser technology may improve snoring by modulating the tone and tensile properties of collagen within the oropharynx and adjacent tissues through photon-mediated activity by PBM [162,170]. PBM also modulates systemic inflammation, reducing pro-inflammatory cytokines and affecting the BBB, immune microenvironment, and T-cell migration [157,160,162,165].

Six weeks of non-invasive PBM in APP/PS1 mice increased mitochondrial energy metabolism and enhanced Aβ clearance by microglia. This prevents cognitive decline by polarizing M1 toward an anti-inflammatory M2 phenotype [171]. PBM devices are readily available and represent a safe technology capable of inducing neuroprotective and anti-inflammatory effects in AD. Pioneering studies have highlighted the use of PBM to enhance brain drainage and facilitate Aβ clearance via meningeal lymphatic vessels, thereby improving cognitive impairment in AD [168]. In a recent nonpharmacological clinical trial (KCT0011155) involving 80 patients with AD, the transcranial PBM (tPBM) technique improved mitochondrial metabolism, cerebral perfusion, and synaptic efficiency. Patients self-administered a tPBM device emitting 808 nm near-infrared light over the bilateral dorsolateral prefrontal cortex (six times per week) for 12 weeks and reported beneficial effects in AD. The results demonstrated that 12 weeks of tPBM treatment was safe and significantly improved cognitive function in AD patients [170,171].

## 8. Conclusions and Future Perspectives

This review studies the role of innate and adaptive immune systems in AD neuropathology. These systems contribute to neurodegeneration in AD models and patients. Chemokines mediate the recruitment of peripheral infiltrates (e.g., monocytes, T and B lymphocytes) into the brain parenchyma and regulate crosstalk between T cells, neurons, and microglia in AD models. Thus, understanding how chemokines drive neuroinflammation could help to develop drugs that target T-cell infiltration into the brain parenchyma of AD models. In fact, CD4^+^ and CD8^+^ T-cell infiltration increases the deposition of Aβ plaques and tau tangles in a chemokine-dependent manner. CXCR6 and CXCR3 signaling orchestrates the recruitment of CD8^+^ T cells into the brain and limits mouse AD pathology. Several chemokines, such as CXCR6-CXCL16 and CXCR3/CXCL10, are potential targets for drugs against AD. Conversely, depleting CD8^+^ T cells and activating Treg cells could induce neuroprotective effects in AD models.

Together, T cells play dual protective and detrimental roles in the brains of rodent AD models. Finally, a pharmacological approach, such as chemokine antagonist treatment, requires more clinical evidence in AD patients. Complementary and emergent strategies, such as PBM, are other future strategies for treating AD in patients.

## Figures and Tables

**Table 1:** Function of chemokine axis.

Cite/Major Cell Type	Functional Role in AD	Chemokine Axis
[61] Endothelial cells	Neuroprotective or neurodegenerative role	Fractalkine = CX3CL1/CX3CR1
[16] Neurons and microglia		
	Fractalkine regulates microglia activation	
	Removal of deposition of beta amyloid deposits in AD models and AD patients	
[4] Neurons	CCR2 is linked to increased amyloid deposits (humans)	CXCR2/CXCL8
[7] Recruitment of peripheral monocytes to plaques into the parenchyma in AD brain	BBB breakdown	CCL2 = MCP-1/CCR2
	Neurodegeneration	
	(AD patients and AD models)	
Produced by astrocytes or microglia cells		
[4] Produced by neurons, astrocytes, and microglia	Promotes monocyte infiltration in the AD brain	CCL3
	Potent chemoattractant, drawing reactive microglia and astrocytes to areas with amyloid-beta plaques	
	CCR5—CCL3 axis facilitates the aggregation of amyloid plaques	
[4,7]	CXCL10 co-localizes with Aβ plaques in APP-transgenic mice	CXCL10/CXCR3
	CXCL10/CXCR3 axis is a major driver of neuroinflammation	
Activates astrocytes	Responses in AD	
	(AD patients)	
	Neurodegeneration	
	Impairs memory formation in LTP	
	(AD models)	
[66] Astrocytes and microglia overexpress CXCL10 and bind to CXCR3 on infiltrating CD8^+^ T cells in the brain	CXCR3 activation recruits T cells in the brain of AD models and AD patients	
[69] The CXCR4/SDF1 alpha is expressed by endothelial cells, neurons, astrocytes, and microglia	Regulate gut dysbiosis and recruit T cells into the brain	SDF 1 Alpha = CXCL12/CXCR4
	Cognitive dysfunction and memory loss in AD	
	Promotes synaptic plasticity and neurogenesis	
	(AD rodent models)	
	Modulates GABAergic inhibition, affecting the balance between excitation and inhibition	
	(AD models)	
[67] CCL5 is mainly secreted by T cells, and it is expressed by platelets, macrophages, and neurons	Neuroinflammation	CCR5 = RANTES/CCR5 axis
	Neuroprotection	
CCR2 receptor is found in astrocytes and microglia	Recruitment of T cells and monocytes, amyloid-beta neuropathology	
Endothelial cells, neurons, glia cells	Markers of AD	CCL19, CCL20
	Neurodegeneration	
	Induces T-cell migration into the brain in AD models	
	CCL24: fluid biomarker for AD progression	
Neurons and glia, lymphoid organs	Critical players of immune cell trafficking in AD	
	The CCL19/CCL21-CCR7 axis mediates both peripheral immune cell homing and brain surveillance	
	Cognitive decline and amyloid-beta pathology	
	Homeostatic chemokines mobilize dendritic cells	
	T cells and mature DCs to lymphoid organs	

**Table 2:** Mean studies with T cells in AD (rodent models and AD patients).

Experimental Model	Immune Cell Subtype	Key Signaling Pathways and Protective vs. Detrimental Effects in AD
Single-cell RNA and T cell receptor (TCR) sequencing of 99,625 high- quality immune cells from 57 leptomeninges and brain samples from donors with AD in patients	Clonal CD8^+^ T cell brain and leptomeningeal immune cells coordinate their activities in AD	The degree of CD8 T_RM_ clonal expansion is positively correlated with microgliaTGFB2 Protective: new opportunities for developing Biomarkers [83]
5xFAD mouse model of amyloidosis	T-cell infiltration in the brain of AD	T-cell infiltration induces cognitive decline in AD Pathogenic: pro-inflammatory cytokine release and exhaustion markers expressing CXCR6^+^ CD39^+^CD73^+/−^ CD8^+^ TRM-like cells. The CD8^+^ T cells overactive microglia around Aβ plaques in the brain of mic [43]
Mouse AD brains	CXCR6 orchestrates brain CD8^+^ T-cell recruitment	CXCR6 orchestrates brain CD8^+^ T-cell residency and limits mouse Alzheimer’s disease pathology. Ligand–receptor interaction by CXCL16–CXCR6 signaling modulates intercellular communication between microglia and CD8^+^ T cells Brain-resident CD8^+^ T cells that coexpress CXCR6 and PD-1 and are in proximity to plaque-associated microglia human and mouse AD brains Protective roles for brain CD8^+^ T cells and CXCR6 in mouse AD pathogenesis [113]
PBMC (Peripheral Blood Mononuclear Cells) T cells from AD patients versus controls (without neurodegeneration), but not of B cells controls.	Telomere length shortening	Telomere length shortening of T cells, but not of B cells or monocytes, correlated with AD status, in the mini mental scores as index of cognitive disfunction Neurodegeneration: T-cell telomere length inversely correlated with serum TNF alpha levels, with apoptosis as well as with the proportion of CD8^+^ T cells lacking expression of the CD28—a costimulatory molecule [92]
Cross-sectional analyses of blood AD and CSF from early AD stages by high-dimensional mass cytometry, single-cell RNA sequencing, ex vivo T-cell secretome analysis, and antigen presentation assays	Altered T-cell reactivity in the early stages of disease	Minor cognitive impairment is associated with increased frequencies of CD8^+^ TEMRA/effector cells in the periphery by inflammatory mediators, and decreased antigen responsiveness It may be beneficial to promote specific CD4^+^ T-cell responses in the preclinical stage of AD [115]
PSAPP AD transgenic mice, were crossbred with the recombination activating gene-2 knockout (Rag2 ko) mice lacking functional B and T cells	The lack of functional B and T cells decreased β -amyloid pathology in AD	Protective: Reduced β-amyloid pathology in an APP AD model lacking functional B and T cells [104]
Comparison of peripheral immune changes in patients with AD mild impairment (MCI) or dementia as compared to controls (without cognitive impairment) by cytometry by time-of-flight CyTOF)	PD1^+^ CD57^+^ CD8^+^ T effect cells for memory cells re-expressing CD45RA in the MCI stage of AD	Neurodegeneration: several innate and adaptive immune cell subsets correlated to CSF biomarkers and cognitive decline in AD Intriguingly, subsets of memory T and B cells were negatively associated with CSF biomarkers for pathology [98]

## Data Availability

No new data were created or analyzed in this study. Data sharing is not applicable.

## Abbreviations

Abeta 42	Amyloid-beta 42 protein
AD	Alzheimer’s disease
APC	Antigen-presenting cell
APP/PS1 transgenic mice	Alzheimer animal model that coexpress two mutated human genes as Amyloid Precursor Protein (APP) and Presenilin 1 (PS1)
ATP	Adenosine Triphosphate
BBB	Blood–brain barrier
CCL2 = MCP-1	Monocyte chemoattractant protein-1
CCL3	Chemokine Ligand 3, also known as Macrophage Inflammatory Pro
CCL5 = RANTES	Regulated on activation, normal T-cell expressed and secreted cells
GHK-Cu	Copper tripeptide
CSF	Cerebrospinal fluid
DAM	Activated, dysfunctional “disease-associated microglia”
DAMP	Disease-associated metabolic profile
GFAP	Glial fibrillary acidic protein
ICAM-1	Intercellular adhesion molecule-1
IGSs	Interferon-stimulated genes
IL-1β	Interleukin-1β
LED	Light-Emitting Diode
LFA-1	Leucocyte function-associated antigen
LTP	Long-term potentiation
MCI	Mild cognitive impairment
MHC	Major histocompatibility antigen
MMPs	Metalloproteases
Nfl	Neurophilament M
NF-κB	Nuclear factor kappa-light-chain-enhancer of activated B cells
NGAL/LNC-2/Lipocalin-2	Neutrophil gelatinase-associated lipocalin
NIR	Infrared near range
NK	Natural Killer
NLRP3	Inflammasome
PBM	Photobiomodulation
PVMs	Perivascular macrophages
ROS	Reactive oxygen species
SOD	Superoxide dismutase
Tregs	Regulatory T cells
TCR	T-cell-receptor cross-recognition
TEMRA CD45RA cells	Effector Memory T cells re-expressing CD45RA
TEMRA CD8 cells	Terminally Differentiated Effector Memory CD8 cells
TNF alpha	Tumor necrosis factor alpha
tPBM	Transcranial photobiomodulation
VCAM-1	Vascular cell adhesion molecule 1
ZO-1	Zonula occludens-1
